# Male Contraception – A Molecular Approach

**DOI:** 10.4103/0970-0218.69283

**Published:** 2010-07

**Authors:** Manjit Panigrahi, Ansuman Panigrahi

**Affiliations:** Animal Genetics Division, Indian Veterinary Research Institute, Izatnagar, Bareilly, Uttar Pradesh, Department of Community Medicine, Kalinga Institute of Medical Sciences, KIIT University, Bhubaneswar, India. E-mail: dr.ansuman@rediffmail.com

Sir,

Population explosion is considered to be one of the hindrances in the development of a nation. This is the reason why India has become the hunger capital of the world in spite of its fast growing economy. In this context, national family welfare programme was launched with the sole aim of controlling the rapidly growing population, promoting health and welfare of the community and thereby contributing to the social development of our nation. Although men and women are considered to share comparable responsibility in family planning and birth control, the primary role of prevention of pregnancy is, to a large extent, borne by women. This situation may, in part, be due to social, cultural, and/or economic factors and poor understanding of factors controlling male fertility. The most important determinant is, perhaps, the limited range of contraceptive options available for men compared with women. Considering the essential roles of several steroid and peptide hormones in spermatogenesis, several studies have focused on suppression of sperm production in the testis by hormonal methods. But it has several drawbacks for which there is a growing interest in the development of non-hormonal methods for male contraceptives and several approaches are being pursued at present.

Almost all adult males produce thousands of spermatozoa each second. After ejaculation, sperms require a period of incubation in the female reproductive tract to fertilize the egg. Upon release into the female reproductive tract, the ejaculated sperms “awaken” to begin a series of biochemical transformations, collectively known as capacitation. Upon contact with the glycoproteins on the surface of the egg, the sperm undergoes the acrosome reaction resulting in the fusion of the plasma membrane and the outer acrosomal membrane and the release of stored hydrolytic enzymes. Once the ovum fuses with a single sperm cell, its cell membrane changes, preventing fusion with other sperm and after the first sperm passes through the zona pellucida of the ovum, the glycoprotein structure of the zona pellucida is altered which is known as the “cortical reaction”.(1)Penetration through the zona pellucida layer of the oocyte requires the sperm to swim in a hyperactivated state at the time and site of fertilization. Studies have indicated that Ca^2+^serves as a key regulator in the initiation and maintenance of motility, including the hyperactivated motility.(2)It has now been discovered that a protein is responsible for sperms’ forceful swimming movements. Normal sperms, which have this protein, beat their tails energetically and show progressive movements. Those lacking it swim with greatly reduced speed and move more randomly. Since the discovery of this unique sperm cation channel-like protein family, named CatSper (Cation channel of Sperm), idea of Ca^2+^requirement for hyperactivation of sperm has been boosted.(3)Four CatSper proteins are required for sperm to form the flagellar ion channels that provide the route of entry for the Ca^2+^.(4)It has been found that the gene product of these four members is expressed exclusively in the testis and differentially localized in the principal piece of sperm tail. It had been observed that targeted disruption of these CatSper proteins led to an identical phenotype similar to that of a normal mouse in which spermatozoa failed to exhibit the hyperactive movement (whip-like flagellar beats). CatSper deficient mice, although infertile, produced normal amount of sperms and exhibited normal sexual behavior. However, they had immotile sperm that could only fertilize eggs without the zona pellucida (but not intact eggs), suggesting that CatSper may be necessary for egg penetration.(5)

Attention to ion channels as drug targets for contraception has grown with the realization that these channel subunits are localized exclusively in sperm and selective knockdown of these subunits can lead to infertility without untoward effects with the rationale that selective inhibitors and/or openers of ion channels could interfere with sperm function.(6)CatSper blocker could also be used by women as it could get from her bloodstream to fluids in the vagina and uterus in time to block the progress of sperm. Preferably, such a drug would be taken before sex. A CatSper blocker would have fewer undesirable side-effects than birth control pills now taken by women because the protein is present only in sperm. Existing pills for women, and many of those under-development for men, contain hormones that effect cells in almost every part of the body. Since CatSper appears to be located only in one section of the sperm tail, drugs targeting it would likely to have only a “much localized effect”.

Almost 60 years have elapsed since the launch of National Family Welfare Programme and still only half of the eligible couples in our country are using various contraceptive methods. This is mainly attributed to lack of information, negative attitude, fear of adverse effects and social influences. As a result, most of the women in reproductive age group bear the burden of unwanted pregnancies and go for abortion, which is mainly unsafe and associated with a high morbidity and mortality. Thus it is highly imperative that in addition to increasing the level of awareness among public regarding different existing contraceptive methods, newer male contraceptives that are safe, effective and acceptable must be developed which could be possible by using CatSper blocker with the help of genetic maneuver so that more males could participate in family planning and birth control as the range of contraceptive options would become wider.
